# "Concentration" and the Future of Medical Education in London

**Published:** 1909-09-04

**Authors:** 


### "CONCENTRATION" AND THE FUTURE OF MEDICAL EDUCATION IN LONDON.

Nothwithstanding the fact that the clinical field offered by the hospitals of the metropolis is still unrivalled, the pre-eminence of its teachers undoubted, it is nevertheless true that more than half the medical schools of London are in financial difficulties. At one time the post of lecturer in a medical school was sufficiently lucrative to make it sought after for its own sake. Nowadays, far from being lucrative, in many of the schools the lecturers are unpaid and the indirect advantages so uncertain that the duties are undertaken only with reluctance and are felt as a burden. The causes of the critical condition of the schools are evident enough. The effects are felt most hardly by the smaller institutions, but are by no means confined to them. The state of things now hurrying to a head was foreseen many years ago, and is the natural outcome of the changed conditions of medical education. For the most part the schools developed out of the system in vogue a hundred years ago of attaching "cubs" or "pups" to individual members of hospital staffs who imparted instruction in every branch of knowledge judged by them necessary to the education of a doctor. With the increasing number, diversity, and complexity of studies impressed upon a student, it soon became necessary to apportion the teaching of different subjects to special teachers, who, however, were still almost exclusively members of the medical staffs. In time, for the whole of the ancillary subjects, it became necessary that the teachers should be men devoted entirely or almost entirely each to his own subject, and so soon as this crystallised, the expenses of a student's education became enormously increased because a body of men was employed whose whole living —however inadequate—had to be made out of teaching. Once this state of things was reached it was obvious that the idea of trying to maintain eleven different sets of teachers in metropolitan schools of medicine, at a time when a great many schools of pure and applied science had sprung up, at a time too when the number of students entering the profession was falling off and when the number of those coming to London was diminishing out of proportion even to this decrease, was unsound economy. Lord Cowper's Commission of 1892, the Statutory Commission of 1898, and Lord Selborne's of 1899 all recognised the fundamental insecurity of the position, and one and all recommended as a way out of the *impasse* the idea of "concentration" among the schools, at any rate for the teaching of the preliminary subjects.

The growth of the provincial schools of medicine, many of them the nuclei of the provincial universities, supported to some extent by State grants and not a few of them endowed by private benefactions has greatly increased the difficulties of the metropolitan problem. Chairs in the provincial schools are so much better endowed than those even in the best-paid London posts that teachers are regularly attracted from London. Conditions as to space both for buildings and for recreation grounds are so infinitely superior in the great provincial cities that subsidiary attractions alone are sufficient to attract many students thither, who in years past would certainly have come to London, and who even now come in large numbers to London for their examinations. The University of London is unique in permitting its degrees to be taken by students who have never spent a day within the walls of any one of its schools.

Another very serious disadvantage of medical education in London is the difficulty presented by the early examinations of the University; or rather, the unsuitability of the earliest examination to the education provided in the schools from which most of the students are drawn. The majority of students educated in the schools of the London University never get the degree. It is not because they do not pass a qualifying examination of the same standard. It is very generally acknowledged that the practical clinical work of the final Conjoint examination is little, if any, inferior to that of the M.B., B.S.London, and we believe that in some respects it surpasses the latter examination. And certainly the standard will compare well with that of the provincial degree examinations. So that the London man is left without any claim to that title of "Doctor" which has so large a commercial value, erroneous though the popular idea of its value may be.

There is some slight hope that this difficulty—this anomaly—may be removed by the legislation following on the report of the present Commission on University Education in London, but it is to be feared that it will only be accomplished by drastic means, and it is not beyond the bounds of possibility that before it comes, simplification will have been attained by the decease from inanition of some of the warring factors of opposition.

There remains just the chance that the traditions of Medical Education in London may be completely preserved and even afforded greater scope than at any time in the history of the metropolitan schools. We have got the material, we have got the teachers, and we believe we can get back the men if it should be found possible to sink jealousies, to bury suspicions and boldly to concentrate. The experiment to this end made by three of the smaller schools has worked without catastrophe, and to their mutual satisfaction, so far as we are aware, for five years, a fact that should encourage others in a similar course.

If for a moment we may be permitted to picture what would be the outcome of a greater degree of concentration; if for example three or four schools would share the expenses of advertisement, share the expenses of providing everything in the way of the indispensable sporting attractions for their men, what economies of time, energy, and money might they not effect at the same time? What a stimulating effect it would have upon the teaching staff if it were known that the students had choice of several hospitals for their clinical instruction! What a good effect upon the students if it were known that the clinical appointments and resident posts were open to competition among the whole of the students of the three or four or five hospitals. What a saving in the energies of lecturers might eventually be secured! What is the difficulty in the way of all this? It probably lies in the fact that the only reward a member of the staff of a London hospital can look for is in the practice he acquires as a consultant to his old pupils. Year by year the work required of him in hospitals has become heavier and heavier; year by year he has turned out men better and better educated, less and less in need of consulting opinion; he is killing by slow starvation the goose that laid his golden eggs, so that to-day it is much harder to make a living than it was twenty or even ten years ago. The gain or loss of students then may be to the members of a hospital staff the saving or loss of their very existence, and it is not to be wondered at that they view radical changes with distrust.

There are many other features that make the problem of medical education difficult. Many factors are contributing to diminish the amount of clinical material, or at least to decentralise it. The medical student of to-day must pursue his fever cases into the suburbs, his cases of mental disease into the country; soon he may be required to look for tuberculosis in special clinics, and special departments of his study in hospitals devoted to small branches of practice. It will not make for his "education" that his instruction should be imparted in fragments. But these difficulties are due to high considerations of public safety, and so long as the growing tendency to municipalisation of control in matters of health and disease is not accompanied by want of consideration for the needs of medical education, no complaint can be made. It is a most significant fact, and one hardly to be appreciated by the public, that, in spite of a cloud of difficulties, London schools—and not alone the great ones—continue to turn out men whose education is not surpassed anywhere in the kingdom; men whose practical knowledge is greater even by reason of the greater individual attention their smaller numbers have enabled them to obtain; and, needless to say, men imbued with the spirit of the best traditions of long generations of great physicians.

One further result of the diminution in the numbers of elementary students has been the development in almost all the London schools of opportunities for the pursuit of post-graduate instruction. More than any other form is this teaching destructive of the sources of income of the teachers, but it is, of course, greatly to the interests of the public that their medical attendants should attain the highest possible skill in every branch of their work, and should be able to revise and renew their knowledge from time to time throughout the period of their lifework. "The greater part of the higher education in England is eleemosynary," has said Sir Edward Fry, and he meant by that "endowed." With the most insignificant exceptions, this is not true of medical education so far as "endowment" is concerned in London; on the other hand, it is increasingly true every year so far as the fact goes that the reward of the great majority of the teaching in London is not only not paid directly, or very inadequately paid, but paid more and more problematically by nebulous indirect means. In London the instruction and inspiration of the future practitioners is a free gift from the teacher to the public. So far the public has shown but very slight recognition of it, and still less encouragement.

